# Subgroup-Independent Mapping of Renal Cell Carcinoma—Machine Learning Reveals Prognostic Mitochondrial Gene Signature Beyond Histopathologic Boundaries

**DOI:** 10.3389/fonc.2021.621278

**Published:** 2021-03-15

**Authors:** André Marquardt, Antonio Giovanni Solimando, Alexander Kerscher, Max Bittrich, Charis Kalogirou, Hubert Kübler, Andreas Rosenwald, Ralf Bargou, Philip Kollmannsberger, Bastian Schilling, Svenja Meierjohann, Markus Krebs

**Affiliations:** ^1^Comprehensive Cancer Center Mainfranken, University Hospital Würzburg, Würzburg, Germany; ^2^Institute of Pathology, University of Würzburg, Würzburg, Germany; ^3^Interdisciplinary Center for Clinical Research, University Hospital Würzburg, Würzburg, Germany; ^4^Guido Baccelli Unit of Internal Medicine, Department of Biomedical Sciences and Human Oncology, School of Medicine, Aldo Moro University of Bari, Bari, Italy; ^5^IRCCS Istituto Tumori “Giovanni Paolo II” of Bari, Bari, Italy; ^6^Department of Internal Medicine II, University Hospital Würzburg, Würzburg, Germany; ^7^Department of Urology and Pediatric Urology, University Hospital Würzburg, Würzburg, Germany; ^8^Center for Computational and Theoretical Biology, University of Würzburg, Würzburg, Germany; ^9^Department of Dermatology, University Hospital Würzburg, Würzburg, Germany

**Keywords:** kidney cancer, pan-RCC, machine learning, mitochondrial DNA, mtDNA, mTOR

## Abstract

**Background:** Renal cell carcinoma (RCC) is divided into three major histopathologic groups—clear cell (ccRCC), papillary (pRCC) and chromophobe RCC (chRCC). We performed a comprehensive re-analysis of publicly available RCC datasets from the TCGA (The Cancer Genome Atlas) database, thereby combining samples from all three subgroups, for an exploratory transcriptome profiling of RCC subgroups.

**Materials and Methods:** We used FPKM (fragments per kilobase per million) files derived from the ccRCC, pRCC and chRCC cohorts of the TCGA database, representing transcriptomic data of 891 patients. Using principal component analysis, we visualized datasets as t-SNE plot for cluster detection. Clusters were characterized by machine learning, resulting gene signatures were validated by correlation analyses in the TCGA dataset and three external datasets (ICGC RECA-EU, CPTAC-3-Kidney, and GSE157256).

**Results:** Many RCC samples co-clustered according to histopathology. However, a substantial number of samples clustered independently from histopathologic origin (*mixed subgroup*)—demonstrating divergence between histopathology and transcriptomic data. Further analyses of *mixed subgroup* via machine learning revealed a predominant mitochondrial gene signature—a trait previously known for chRCC—across all histopathologic subgroups. Additionally, ccRCC samples from *mixed subgroup* presented an inverse correlation of mitochondrial and angiogenesis-related genes in the TCGA and in three external validation cohorts. Moreover, *mixed subgroup* affiliation was associated with a highly significant shorter overall survival for patients with ccRCC—and a highly significant longer overall survival for chRCC patients.

**Conclusions:** Pan-RCC clustering according to RNA-sequencing data revealed a distinct histology-independent subgroup characterized by strengthened mitochondrial and weakened angiogenesis-related gene signatures. Moreover, affiliation to *mixed subgroup* went along with a significantly shorter overall survival for ccRCC and a longer overall survival for chRCC patients. Further research could offer a therapy stratification by specifically addressing the mitochondrial metabolism of such tumors and its microenvironment.

## Introduction

Basic and clinical research in renal cell carcinoma (RCC) mainly focuses on established histopathologic subgroups, specifically clear cell (ccRCC), papillary (pRCC) and chromophobe RCC (chRCC). Accordingly, histopathology is of crucial relevance for determining treatment strategies including drug sequencing in RCC patients, especially in a metastasized situation. As reflected in the WHO classification for renal neoplasms (1), dividing RCC in three distinct (sub-)entities does not completely mirror tumor biology and its complexity. Instead, sub-categories such as clear cell papillary RCC (2) were introduced, indicating substantial greyscales between classical histopathologic subgroups.

By performing transcriptomic analyses, researchers have identified characteristic signatures of ccRCC, pRCC, and chRCC—thereby supporting established histopathologic classification (3–5). Although comprehensive pan-RCC analyses have been performed previously, the boundaries of histopathologic origin usually were not scrutinized (6, 7).

Using principal component analysis (PCA) with subsequent machine learning (ML) algorithms, we mapped 891 RCC specimen irrespective of histopathologic boundaries. Following this comprehensive pan-RCC approach allowed us to identify novel RCC subgroups with a prognostic impact for cancer patients and provide first functional insight.

## Materials and Methods

### Data Acquisition

This work mainly based on data provided by The Cancer Genome Atlas (TCGA) consortium. Utilized entities were ccRCC (KIRC cohort, *n* = 538 tumor samples), chRCC (KICH cohort, *n* = 65 samples) and pRCC (KIRP cohort, *n* = 288 samples) downloaded from the GDC portal (https://portal.gdc.cancer.gov). For evaluation, we used data provided by the ICGC (international network of cancer genome projects) (8), specifically the RECA-EU data set, comprising of *n* = 91 ccRCC samples (https://dcc.icgc.org/projects/RECA-EU) with available RNA-sequencing data. Additionally, we used ccRCC samples from the CPTAC-3-Kidney cohort (*n* = 101) as further external validation (https://portal.gdc.cancer.gov/projects/CPTAC-3) (9). Regarding RCC caused by hereditary leiomyomatosis (hlRCC)—also known as fumarate hydratase (FH)-deficient RCC—we further examined the smaller GSE157256 cohort (10) as another source for evaluation (*n* = 26) (https://www.ncbi.nlm.nih.gov/geo/query/acc.cgi?acc=GSE157256). For all mentioned datasets, we used unprocessed FPKM or RSEM (GSE157256) values as provided.

### Bioinformatical Analyses

The presented work was implemented in a Jupyter Notebook environment (version 7.5.0)—which is available upon request—using the Python version 3.6.9, SciPy version 1.3.0 (11) and scikit-learn version 0.22.1 (12).

### T-SNE Plotting

Our project was based on the 2D representation of high-dimensional data with subsequent cluster analysis using ML. For plotting unprocessed FPKM data in 2D, we performed a PCA with 50 components—using PCA of the sklearn.decomposition module—and used the results as input for t-SNE plotting (sklearn.manifold module) (13). For calculating and plotting in 2D, we used a random initiation with a learning rate of 300 and a perplexity of 27 with 10.000 iterations. For reproducibility, we used the random state 0 (n_components=2, init='random', perplexity=27, n_iter=10,000, learning_rate=300, random_state=0). Additionally, cluster annotation and t-SNE coordinates for each TCGA sample from all RCC subgroups are shown in Supplementary Table 1.

### Random Forest Learning

After manual annotation, we used these clusters for subsequent learning steps. For this, we applied a model utilizing Random Forest (RF) Classifier (RandomForestClassifier of the sklearn.ensemble module). For training our model, we used a 70/30 split, letting the model learn on 70% of the data and evaluating it on the remaining 30%, with 1,000 trees in the forest (n_estimators=1,000), leaving out the pRCC samples not clustering in one of the three annotated clusters or the *mixed subgroup*. For further investigation, we trained 20 models and used the one with the highest test accuracy for subsequent feature analysis. For this purpose, we assigned the according “feature values,” implying the importance of each feature, to each feature, representing the Ensembl gene IDs. We identified the top 200 genes with the strongest influence on our model, which distinguished our manually annotated clusters with the highest accuracy. These top 200 genes of our best performing model overlapped in 92 genes with the mean of the other 19 trained models, outperforming them in test accuracy—with 92.06% compared to the mean of 83.42% (min. 79.37%, max. 86.11%). A 10-fold-crossvalidation of the data yielded a mean accuracy of 84.52% (+/- 9.16%).

### Plots and Statistical Analysis

Correlation and scatter plots were generated using matplotlib. Indicated Pearson Rs were calculated using the according module from scipy.stats. For subsequent survival analysis of patient survival, Kaplan Meier (KM) plots were generated using the lifelines module (version 0.23.1) with the KaplanMeierFitter (14).

If not stated otherwise, statistical tests were performed using Kruskal-Wallis-Test—using scipy.stats module including indicated significances, for which we used the statannot module for python (version 0.2.3; https://github.com/webermarcolivier/statannot). For the analysis of further interactions and relations between the identified 200 genes with the highest influence on the learned model, we used a Network generated by StringDB (15). The coloring of the nodes was done directly by StringDB for selected gene-sets stated as significant.

For validation purposes of relevant pathways and genes previously identified by mRNA expression patterns, we used the level four protein expression levels provided by The Cancer Proteome Atlas (16) (TCPA—https://tcpaportal.org/tcpa/) for the three investigated cohorts.

## Results

### Clustering 891 RCC Samples Independently From Histopathologic Origin

**Figure 1:**
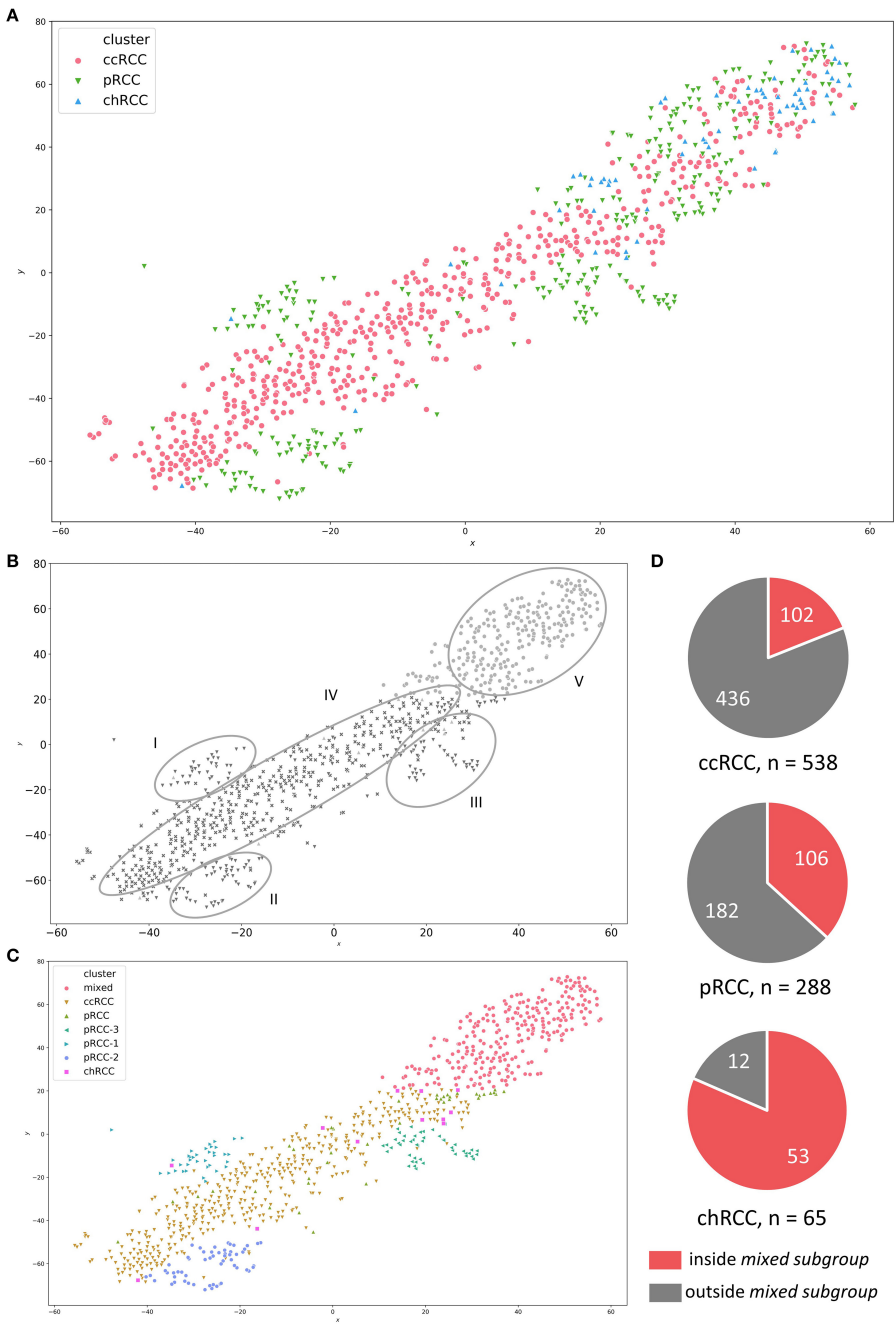
**(A)** t-SNE-plot for RNA-sequencing data from ccRCC (red), pRCC (green) and chRCC (blue) specimen within the TCGA database. **(B)** Visually identified clusters—I to III: distinct pRCC subgroups; IV: ccRCC samples; V: mixed subgroup containing ccRCC, pRCC and chRCC tumors. **(C)** Manually defined clusters based on visual separation. **(D)** Pie charts illustrating absolute numbers and proportions of RCC samples inside/outside the mixed subgroup for each RCC subgroup.

For PCA, we used the RNAseq data of all registered RCC specimen (*n* = 891) within the TCGA database, irrespective of their histopathologic origin. We combined all tumor specimen in a t-SNE-plot in order to illustrate familiarities and discordances between samples based upon unprocessed FPKM values. Figure 1A represents the t-SNE plot, with ccRCC, pRCC and chRCC samples marked in red, green and blue, respectively. Of note, most tumor samples clustered to RCC subgroups, thereby confirming the familiarity and the validity of histopathologic classification. Figure 1B represents the schematic distribution of clusters identified within the t-SNE-plot. Interestingly, pRCC samples did not cluster in a single subgroup, but instead in three distinct subgroups (cluster I-III), whereas ccRCC specimen built another cluster (IV). However, apart from most samples clustering according to histopathology, we identified a distinct cluster containing a mixture of ccRCC, pRCC and chRCC samples (Figure 1B; cluster V). We named this accumulation *mixed subgroup*. As depicted in Figure 1C, we manually split and defined the novel clusters for further ML-based analyses. Aside from three distinct pRCC clusters, which surely merit future investigation, we were mainly interested in this *mixed subgroup*—containing 19% of ccRCC, 36.8% of pRCC and 81.5% of chRCC samples (Figure 1D). Of note, our clustering approach revealed no clear separation between type 1 and type 2 pRCC (Supplementary Figure 1).

### Clinical Characterization of Patient Samples From *Mixed Subgroup*

We next examined the clinical characteristics of RCC patient samples depending on their affiliation to the *mixed subgroup* (Table 1). Comparing ccRCC samples inside and outside the *mixed subgroup*, we found no significant differences in age, gender, tumor stage, tumor extension (T classification), lymphonodal invasion (*N* classification) or metastasis (M classification). In contrast, tumor grading was significantly different (*p* = 0.014). For pRCC, *mixed subgroup* patients were significantly older (65.1 ± 10.9 vs. 59.6 ± 12.1 years; *p* = 0.0002) than patients with pRCC not belonging to this cluster. Moreover, the proportion of male patients was significantly higher in the *mixed subgroup* (*p* = 0.001). In contrast to the age distribution in patients with pRCC, chRCC samples from the *mixed subgroup* were significantly younger (49.6 ± 13.2 vs. 61.9 ± 12.9 years; *p* = 0.012). In addition, the lymphonodal status differed significantly between the two subgroups (*p* = 0.005).

**Table 1:** Clinical characteristics of RCC patients inside and outside the mixed subgroup.

		**ccRCC non-mixed**	**ccRCC mixed**	***p***	**pRCC non-mixed**	**pRCC mixed**	***p***	**chRCC non-mixed**	**chRCC mixed**	***p***
		***n*** **=** **428**	***n*** **=** **102**		***n*** **=** **182**	***n*** **=** **105**		***n*** **=** **12**	***n*** **=** **53**	
**Age**	mean	60.2 ± 2.2	62.0 ± 11.8	0.196	59.6 ± 12.1	65.1 ± 10.9	**0.00021**	61.9 ± 12.9	49.6 ± 13.2	**0.012**
**Gender**	m	273 (63.79%)	71 (69.61%)	0.269	123 (67.58%)	89 (84.76%)	**0.001**	10 (83.33%)	29 (54.72%)	0.07
	f	155 (36.21%)	31 (30.39%)		59 (32.42%)	16 (15.24%)		2 (16.67%)	24 (45.28%)	
**Tumor stage**	I	223 (52.47%)	42 (64.29%)	0.166	108 (64,70%)	64 (38,32%)	0.419	2 (16.67%)	18 (33.96%)	0.1
	II	43 (10.12%)	14 (8.33%)		16 (9,60%)	4 (2,40%)		5 (41.67%)	20 (37.74%)	
	III	90 (21.18%)	33 (19.64%)		34 (20,30%)	16 (9,58%)		1 (8.33%)	13 (24.53%)	
	IV	69 (16.23%)	13 (7.74%)		9 (5,40%)	6 (3,60%)		4 (33.33%)	2 (3.77%)	
**T**	T1	228 (53.27%)	43 (42.57%)	0.092	119 (64.67%)	74 (70.48%)	0.463	2 (16.67%)	18 (33.962%)	0.14
	T2	53 (12.38%)	16 (15.84%)		22 (11.96%)	10 (9.52%)		5 (41.67%)	20 (37.74%)	
	T3	137 (32.00%)	41 (40.59%)		39 (21.20%)	20 (19.05%)		3 (25%)	15 (28.30%)	
	T4	10 (2.33%)	1 (0.99%)		4 (2.17%)	1 (0.95%)		2 (16.66%)	0 (0%)	
**N**	N0	192 (93.66%)	47 (94%)	0.929	29 (59.18%)	20 (71.43%)	0.21	4 (57.14%)	35 (94.60%)	**0.005**
	N1	13 (6.34%)	3 (6%)		16 (32.66%)	8 (28.57%)		2 (28.57%)	1 (2.7%)	
	N2	0 (0%)	0 (0%)		4 (8.16%)	0 (0%)		1 (14.29%)	1 (2.7%)	
**M**	M0	19 (90.48%)	3 (75%)	0.392	60 (63.16%)	35 (89.74%)	0.654	4 (80%)	3 (75%)	0.866
	M1	2 (9.52%)	1 (25%)		35 (36.84%)	4 (10.26%)		1 (20%)	1 (25%)	
**Grading**	G1	13 (3.06%)	13 (11.93%)	**0.014**						
	G2	195 (45.88%)	32 (29.36%)							
	G3	158 (37.18%)	48 (44.04%)							
	G4	59 (13.88%)	16 (14.67%)							

*Except for age (mean ± standard deviation), all characteristics were presented as absolute values. p-values highlighted as bold were significant for p < 0.05*.

### ML-Based Functional Characterization of Patient Samples Affiliated to *Mixed Subgroup*

**Figure 2:**
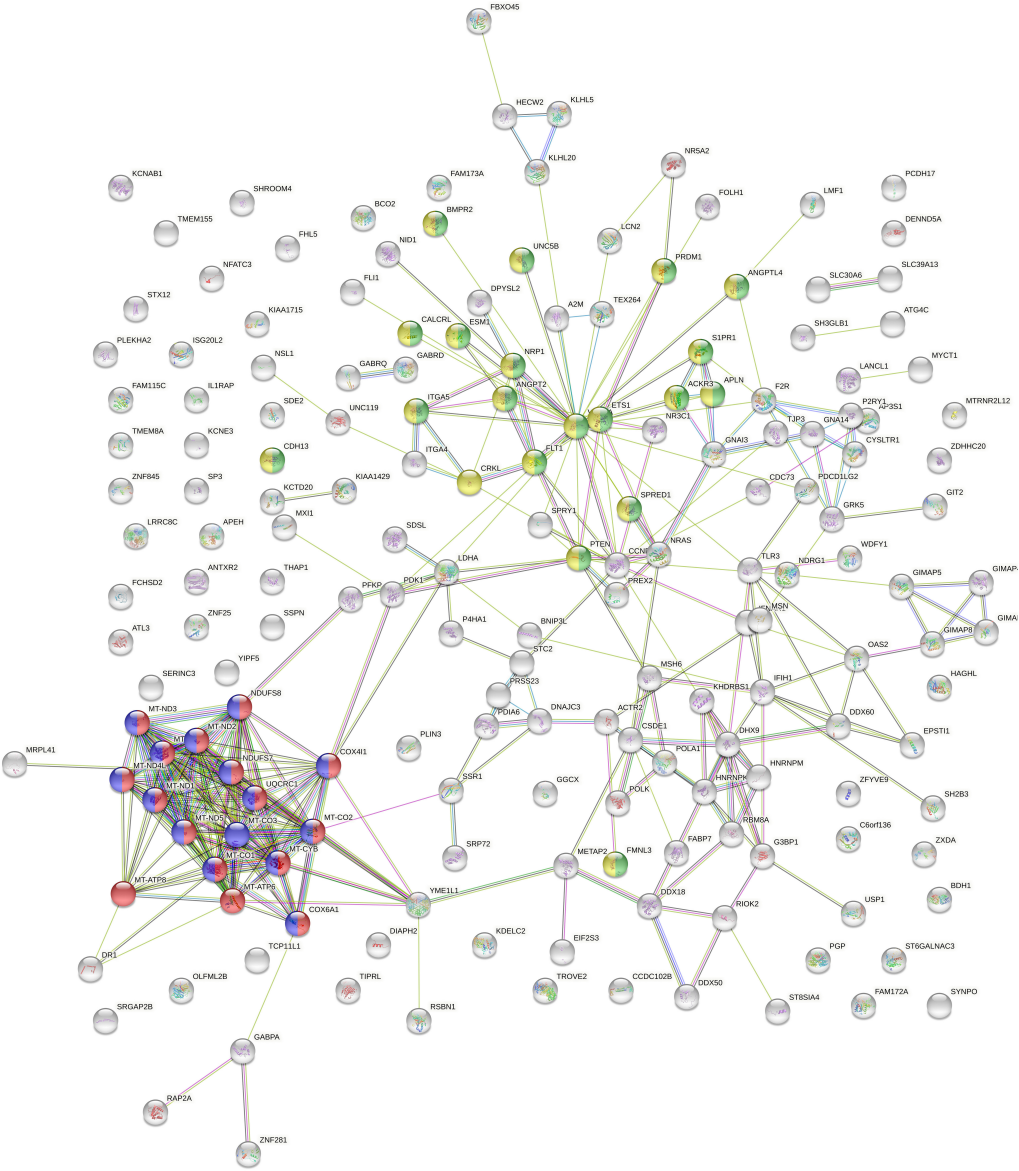
StringDB network of the top 200 genes identified as relevant classifiers for RCC sample clusters from Figure 1C. Genes affiliated with oxidative phosphorylation and respiratory electron transport chain are marked in red and blue, genes related to blood vessel morphogenesis and blood vessel development are marked in green and yellow.

To learn more about functional traits and characteristic differences of the clusters, we applied further ML based on the visual separation (Figure 1C). Therefore, we determined the top 200 genes best classifying the novel clusters. As shown in Figure 2, we depicted these genes in a StringDB gene network to uncover relevant signaling pathways. We found a substantial accumulation of mitochondrial genes—with String DB identifying “oxidative phosphorylation” (GO:0006119) and “respiratory electron transport chain” (GO:0022904) as highly overrepresented pathways in our analysis. Additionally, “blood vessel development” (GO:0001568) and “blood vessel morphogenesis” (GO:0048514) were also highly overrepresented.

Moreover, mtDNA genes represented all of the top 10 classifier genes in our RF calculation—as shown in Table 2. In conclusion, we found mitochondrial and angiogenesis-related gene signatures to be most predictive within our clustering approach.

**Table 2:** Gene families significantly overrepresented in the top 200 cluster classifying genes from Random Forest (RF) analysis.

**Mitochondrial Genes**			**Angiogenesis-related Genes**		
**HGNC Symbol**	**Ensembl gene ID**	**RF-Feature Position**	**HGNC Symbol**	**Ensembl gene ID**	**RF-Feature Position**
MT-CYB	ENSG00000198727	1	ETS1	ENSG00000134954	13
MT-ND4	ENSG00000198886	2	ANGPT2	ENSG00000091879	33
MT-CO1	ENSG00000198804	3	APLN	ENSG00000171388	37
MT-CO3	ENSG00000198938	4	FLT1	ENSG00000102755	38
MT-CO2	ENSG00000198712	5	CRKL	ENSG00000099942	46
MT-ND4L	ENSG00000212907	6	ITGA5	ENSG00000161638	54
MT-ATP6	ENSG00000198899	7	NRP1	ENSG00000099250	56
MT-RNR1	ENSG00000211459	8	PRDM1	ENSG00000057657	93
MTATP6P1	ENSG00000248527	9	PTEN	ENSG00000171862	109
MT-ND1	ENSG00000198888	10	VEGFA	ENSG00000112715	112
MT-ND2	ENSG00000198763	20	ACKR3	ENSG00000144476	114
MT-ND3	ENSG00000198840	24	CDH13	ENSG00000140945	146
MT-RNR2	ENSG00000210082	25	BMPR2	ENSG00000204217	148
			CALCRL	ENSG00000064989	177
			ESM1	ENSG00000164283	191

*For each gene, HGNC symbol, Ensembl gene IDs, and the position in our calculation is shown*.

### Mitochondrial and Angiogenesis-Related Genes Inside and Outside *Mixed Subgroup*

**Figure 3:**
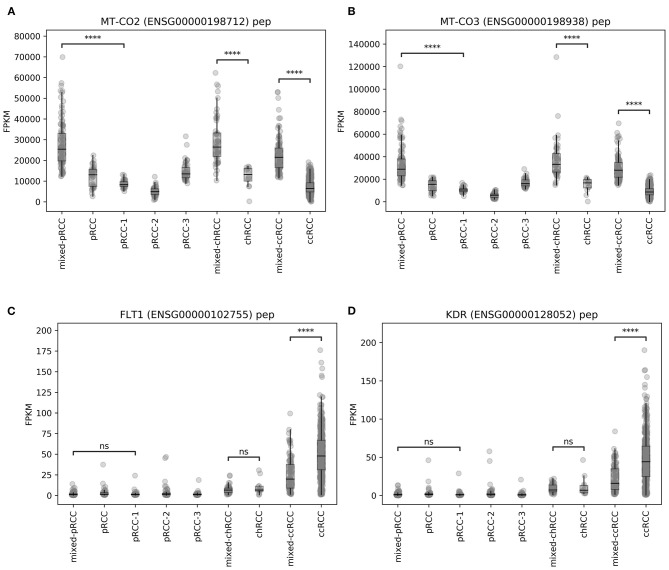
Unprocessed FPKM values of exemplary candidate genes–**(A,B)** MT-CO2 and MT-CO3, **(C,D)** FLT1 and KDR. ns, not significant, ****p < 0.0001.

Alterations and overexpression of mtDNA have been described as characteristic traits of chRCC (5, 17, 18)—and more than 80% of the chRCC samples in our analysis were located in the *mixed subgroup* (see Figure 1D). Due to this relative overrepresentation of chRCC in this cluster, we first checked whether our RF analysis was biased by a high proportion of chRCC samples. For this reason, we compared unprocessed FPKM values of mitochondrial genes for ccRCC, pRCC, and chRCC samples depending on the affiliation to the *mixed subgroup*. We found a highly significant overexpression of mitochondrial genes for chRCC samples inside compared to samples outside the *mixed subgroup*. However, alterations in mtDNA expression were not limited to chRCC. Instead, *mixed subgroup* samples from pRCC as well as ccRCC exhibited a highly significant mtDNA overexpression. Figures 3A,B illustrate unprocessed FPKM values for candidate genes MT-CO2 (Figure 3A) and MT-CO3 (Figure 3B).

**Figure 4:**
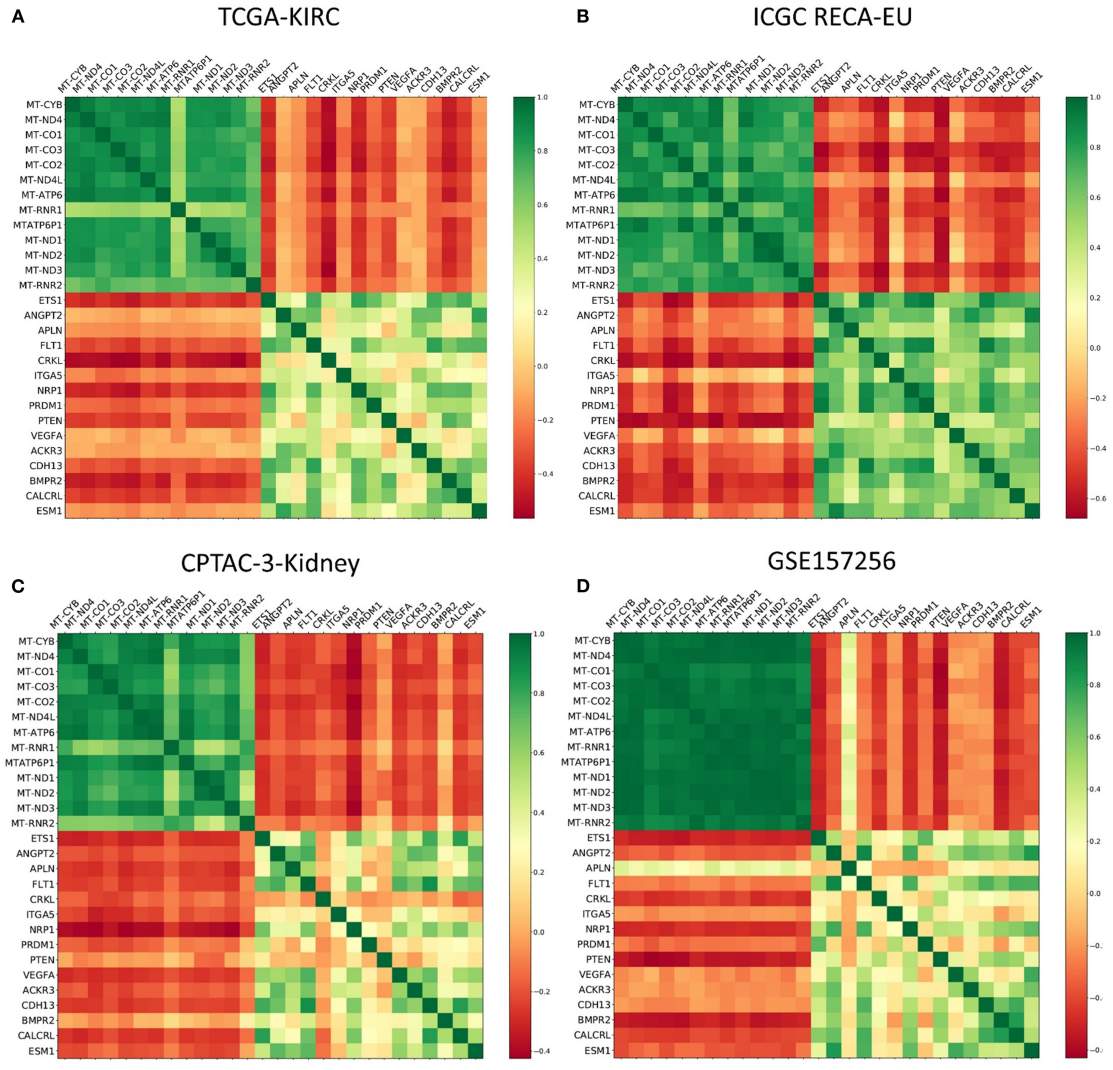
Color-coded presentation of the Pearson R correlation matrix of mitochondrial genes and angiogenesis-associated genes for ccRCC samples from the **(A)** TCGA, **(B)** the ICGC RECA-EU, and **(C)** the CPTAC-3-Kidney cohort as well as **(D)** Fumarate hydratase-deficient RCC samples contained within the GSE157256 cohort.

For angiogenesis-related genes such as FLT1 (Figure 3C) and KDR (Figure 3D), we discovered significantly lower expression levels within ccRCC samples from *mixed subgroup*. Additionally, we discovered significant expression differences for genes displayed in Table 2, regardless of the underlying histopathologic entity, when compared to normal tissue samples (Supplementary Figures 2–7). Regarding expression of mitochondrial and angiogenesis-related genes in ccRCC, we found negative Pearson R correlations in the TCGA dataset (Figure 4A) as well as all three RCC validation cohorts (Figures 4B–D). In line with a weaker angiogenesis signature, ccRCC and pRCC samples from *mixed subgroup* displayed significantly lower levels of c-MET (Supplementary Figure 8).

Summing up the results, mtDNA and angiogenesis signatures proved to be predictive not only for our pan-RCC clustering approach—but also specifically for ccRCC samples. Moreover, expression levels of mitochondrial and angiogenesis-associated genes were negatively correlated in four independent RCC cohorts.

### Impact of *Mixed Subgroup* Affiliation on Patient Survival

**Figure 5:**
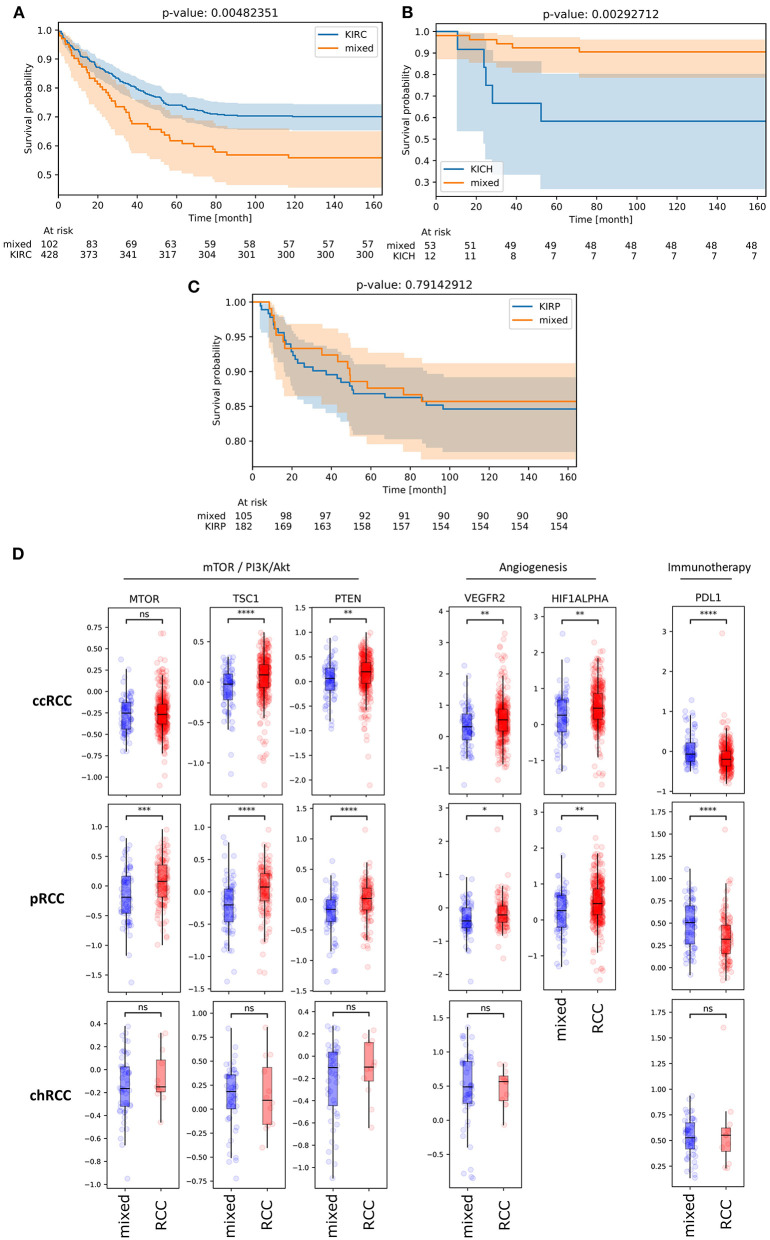
**(A,B)** KM plots illustrating overall survival of patients with ccRCC **(A)**, chRCC **(B)** and pRCC **(C)** from TCGA database depending on mixed subgroup affiliation. **(D)** Protein expression levels of *bona fide* candidate genes from mTOR-associated, angiogenesis-related and immune-related signaling for ccRCC, pRCC and chRCC samples inside (blue) and outside (red) the mixed subgroup (TCPA database). ns, not significant. *p < 0.05, **p < 0.01, ***p < 0.001, ****p < 0.0001.

After characterizing *mixed subgroup* samples from a clinical and a functional perspective, we next investigated whether an affiliation to this cluster impacted patient prognosis. Strikingly, survival analysis revealed a significantly worse prognosis (*p* = 0.005) for ccRCC patients from the TCGA database belonging to the *mixed subgroup* (Figure 5A). For chRCC patients (Figure 5B), cluster affiliation had the opposite effect—with significantly higher survival rates (*p* = 0.003) for patients inside the *mixed subgroup*. In contrast, there was no significant survival difference for patients with pRCC (Figure 5C).

Given that clinical characteristics such as tumor stage and TNM classification did not differ significantly for patients with ccRCC (Table 1), we reasoned that the survival impact could partially be due to an inadequate therapy stratification. Using *The Cancer Proteome Atlas* (TCPA) (16, 19), we therefore analyzed the protein expression of *bona fide* gene candidates related to mTOR and PI3K/Akt signaling, angiogenesis and immune signaling (Figure 5D). Regarding ccRCC as well as pRCC samples, we found a significant downregulation of VEGFR2 and HIF1A protein expression in *mixed subgroup* samples. For both subgroups, this downregulation of angiogenesis-related genes was accompanied by a significant upregulation of PD-L1 expression. Moreover, protein expression of TSC1 and PTEN was downregulated in *mixed subgroup* samples. While pRCC samples from our novel cluster exhibited a significant mTOR downregulation, the slight increase in mTOR protein expression of ccRCC samples from *mixed subgroup* was not significant. Potentially due to lower sample numbers, TCPA analysis revealed no significant expression differences for chRCC specimen.

In summary, we found a highly significant and clinically relevant influence of *mixed subgroup* affiliation in RCC patients from the TCGA database—with a better prognosis for chRCC and a worse overall survival for ccRCC patients.

## Discussion

Classifying cancer tissue into three histopathologic subgroups—clear cell, papillary and chromophobe—critically determines treatment strategies and prognosis of RCC patients. However, growing evidence highlights that this classification is not absolute nor distinct. Instead, the WHO system of renal cell tumors from 2016 contained several additional subgroups, such as succinate dehydrogenase-deficient renal carcinoma and clear cell papillary RCC (1). Previous functional analyses on RCC mainly focused on isolated gene signatures, which were characteristic and prognostic for single histopathologic subgroups (4, 5, 20, 21)—e.g., ClearCode34 (22) for determining the individual risk of recurrence in localized ccRCC. Moreover, researchers aimed to identify biomarkers and gene networks predictive of future therapy response—especially for angiogenesis inhibition, tyrosine kinase inhibition (TKI) and immune checkpoint blockade (23–27). Interestingly, a recent study was able to discriminate ccRCC and pRCC samples originating from proximal tubules of the nephron from chRCC specimen originating from distal tubules based on the metabolic and lipidomic profile of the samples (28).

### Pan-RCC Clustering Identifies Subgroup Beyond Established Histopathologic Classification

While most studies focused on gene signatures within given histopathologic boundaries, we aimed to challenge the absoluteness and robustness of RCC subgroup classification. In our pan-RCC approach, we performed a clustering for all RCC specimen from the TCGA database. Of note, a substantial number of RCC samples clustered independently from histopathologic origin. We called this cluster *mixed subgroup*. Conferring samples inside and outside the *mixed subgroup*, ccRCC patients exhibited no significant differences regarding age, gender, tumor stage and TNM classification. In contrast, grading of tumor samples appeared significantly different, partially due to a higher proportion of G1 tumors in the *mixed subgroup*. pRCC patients from this cluster were significantly older than the remainder of the group. Moreover, the proportion of male patients was higher inside the *mixed subgroup*. All other clinical characteristics did not differ significantly. Patients with chRCC within the cluster were significantly younger and had a higher proportion of N0 patients.

### ML Reveals Mitochondrial Genes as Most Influential for Pan-RCC Clustering

For further functional characterization, we applied RF learning in order to identify gene signatures most predictive for the novel clusters. This ML approach revealed mitochondrial genes to be most influential for the basic clustering, followed by genes related to angiogenesis. As mtDNA overexpression is a reported feature of chRCC (5, 17, 18), we had to rule out a systematic bias caused by the high proportion of chRCC samples within the *mixed subgroup*. Therefore, we analyzed mtDNA expression in all RCC subgroups depending on subgroup affiliation. Of note, ccRCC, pRCC, and chRCC specimen belonging to the *mixed subgroup* all displayed significantly higher levels of mtDNA expression compared to the counterparts outside this cluster. In ccRCC, this mtDNA upregulation went along with a downregulation of angiogenesis-related genes. Taking these results together led to significantly negative correlations between mitochondrial and angiogenesis signatures—not only in ccRCC samples from TCGA but also in the RECA-EU and CPTAC-3-Kidney cohorts taken as external validation. Moreover, comparable results from the GSE157256 cohort representing fumarate hydratase-deficient RCC could imply a general underlying mechanism beyond RCC subgroups.

A pan-RCC subgroup characterized by a prominent mtDNA signature appeared surprising at first sight. Although aberration in mitochondrial signaling is known across RCC subgroups, these deviations are not considered being unidirectional toward an upregulation of mitochondrial transcripts and mitochondrial mass (29). While mtDNA aberrations and overexpression are mainly regarded as a characteristic trait of chRCC tissue (5, 17, 18), downregulation of mitochondrial enzymes with increasing tumor stages and decreased oxidative capacity were previously reported for ccRCC (30–32). However, growing evidence indicates that mtDNA can also have oncogenic functions, thereby appearing as a potential (co-)target in future cancer therapies (33). Specifically, researchers showed that tumor cells lacking mtDNA could not metastasize *in vivo*—after restoration of mtDNA levels, cancer cells regained this ability (34). In line with these findings, Schöpf et al. demonstrated the importance of oxidative phosphorylation in high-grade prostate cancer by describing a high-risk subgroup characterized by a distinct mitochondrial signature (35). Given the established role of angiogenesis and angiogenesis-related genes such as VEGFR2 in high-risk prostate cancer (36, 37), further examining the interaction of mitochondrial and angiogenesis pathways in prostate cancer could prove beneficial.

### Assessing Prognosis and Therapeutic Windows for *Mixed Subgroup* Patients

Importantly, ccRCC patients inside the *mixed subgroup* suffered from significantly worse overall survival. This result was even more surprising given the non-significant differences in TNM stage between both subgroups. For pRCC patients, we did not find significant survival differences regarding *mixed subgroup* affiliation. However, we identified three distinct pRCC clusters. This result surely merits further investigation regarding functions and prognosis of each pRCC cluster. In contrast, patients with chRCC belonging to the *mixed subgroup* exhibited a significantly longer overall survival. In summary, survival data from ccRCC and chRCC patients underline the role of the *mixed subgroup* as a novel prognostic RCC cluster identified by our comprehensive clustering approach. Regarding the striking survival impact in ccRCC combined with non-differing clinical characteristics, it was tempting to assume that diverging outcomes were at least partially treatment-related.

First, addressing mtDNA overexpression appears as an attractive therapeutic approach, as compounds such as the anthelmintic drug atovaquone (38) and the antibiotic tigecycline (39) were shown to chemo-sensitize RCC. Moreover, our results could explain the beneficial effect of additional Metformin intake during treatment of metastatic RCC, as this biguanide works as a mitochondrial inhibitor (40–42). Above all, mTOR inhibitors—which already have the approval for treating RCC—appear as promising candidates for *mixed subgroup* RCC patients. mTOR signaling is tightly linked to mitochondrial function (43, 44). Recently, a mitochondrial complex I inhibitor (IACS-010759) targeting oxidative phosphorylation in cancer cells showed efficacy in brain cancer and leukemia models (45).

Given the downregulation of angiogenesis-related gene signatures in ccRCC patients from the *mixed subgroup*, inhibition of angiogenesis and TKI do not appear as attractive first-line approaches for these patients. Indeed, the predominant use of TKI within the historical RCC cohort from the TCGA database may partially explain the striking survival differences observed in this analysis. Supporting our findings from RNA-sequencing, protein levels of VEGFR2 and HIF2A were lowered in the *mixed subgroup* for ccRCC as well as pRCC patients. Regarding the downregulation of c-MET in ccRCC and pRCC samples from the *mixed subgroup*, treatment with MET (co-)inhibitors such as Cabozantinib (46) does not appear promising, either.

Further research could clarify whether immune checkpoint inhibition constitutes a viable treatment strategy in our new cluster. At first sight, highly significant protein overexpression of PD-L1 in ccRCC and pRCC patients from the *mixed subgroup* makes it an attractive therapeutic target. However, unlike in entities as melanoma and non-small cell lung cancer (47), it is still unclear whether PD-L1 overexpression in RCC results in better response to immunotherapy (48). Completely in line with our findings, several clinical trials already stated that PD-L1 overexpression marked a RCC high-risk cohort (48, 49).

Our study has some limitations regarding its methodology and its retrospective nature. We are aware that manual cluster annotation approaches naturally contain immanent biases. Moreover, our findings derive from the re-analysis of historic cohorts and require further—ideally prospective—validation in future studies. Essentially, we identified a high-risk ccRCC subgroup best described by a mitochondrial signature and a downregulation of angiogenesis-related genes, which was not exclusive to one RCC subgroup. Although preliminary, these results could contribute to an individual risk classifier based on transcriptomic data from patients' samples and help establishing personalized medicine in RCC.

## Data Availability Statement

Publicly available datasets were analyzed in this study. This data can be found here: https://portal.gdc.cancer.gov/projects https://dcc.icgc.org/projects/RECA-EU https://www.ncbi.nlm.nih.gov/geo/query/acc.cgi?acc=GSE157256 https://portal.gdc.cancer.gov/projects/CPTAC-3.

## Author Contributions

AM, AGS, BS, SM, MK: conceptualization. AM, PK: methodology. AM, AGS, AK, MB, MK: writing – draft preparation. AM, AGS, MB, CK, HK, AR, RB, PK, BS, SM, MK: writing – review and editing. HK, AR, RB, BS, SM: supervision/funding/infrastructure. All authors contributed to the article and approved the submitted version.

## Conflict of Interest

The authors declare that the research was conducted in the absence of any commercial or financial relationships that could be construed as a potential conflict of interest.

## Supplementary Material

Supplementary Figure 1 Isolated illustration of pRCC samples from the pan-RCC clustering approach based on t-SNE plotting—with known affiliation to clinically established type 1 and type 2 pRCC subtypes. RCC, renal cell carcinoma; pRCC, papillary renal cell carcinoma.

Supplementary Figure 2 Expression comparison between clear cell renal cell carcinomas outside (ccRCC) and inside (mixed) the *mixed subgroup* and respective normal tissue samples for mitochondrial genes identified by machine learning. ns, not significant. ^*^*p* < 0.05, ^**^*p* < 0.01, ^***^*p* < 0.001, ^****^*p* < 0.0001.

Supplementary Figure 3 Expression comparison between clear cell renal cell carcinomas outside (ccRCC) and inside (mixed) the *mixed subgroup* and respective normal tissue samples for angiogenesis genes identified by machine learning. ns, not significant. ^*^*p* < 0.05, ^**^*p* < 0.01, ^***^*p* < 0.001, ^****^*p* < 0.0001.

Supplementary Figure 4 Expression comparison between the identified papillary renal cell carcinoma cluster outside (pRCC 1 to 3) and inside (mixed) the *mixed subgroup* and respective normal tissue samples for mitochondrial genes identified by machine learning. ns, not significant. ^*^*p* < 0.05, ^**^*p* < 0.01, ^***^*p* < 0.001, ^****^*p* < 0.0001.

Supplementary Figure 5 Expression comparison between the identified papillary renal cell carcinoma cluster outside (pRCC 1 to 3) and inside (mixed) the *mixed subgroup* and respective normal tissue samples for angiogenesis genes identified by machine learning. ns, not significant. ^*^*p* < 0.05, ^**^*p* < 0.01, ^***^*p* < 0.001, ^****^*p* < 0.0001.

Supplementary Figure 6 Expression comparison between chromophobe renal cell carcinomas outside (chRCC) and inside (mixed) the *mixed subgroup* and respective normal tissue samples for mitochondrial genes identified by machine learning. ns, not significant. ^*^*p* < 0.05, ^**^*p* < 0.01, ^***^*p* < 0.001, ^****^*p* < 0.0001.

Supplementary Figure 7 Expression comparison between chromophobe renal cell carcinomas outside (chRCC) and inside (mixed) of *mixed subgroup* and respective normal tissue samples for angiogenesis genes identified by machine learning. ns, not significant. ^*^*p* < 0.05, ^**^*p* < 0.01, ^***^*p* < 0.001, ^****^*p* < 0.0001.

Supplementary Figure 8 Unprocessed FPKM values for c-MET within manually annotated clusters across RCC subgroups. ns, not significant. ^**^*p* < 0.01, ^****^*p* < 0.0001.

Supplementary Table 1 Coordinates within the t-SNE-plot and subsequent attributed cluster affiliation for all RCC samples from the TCGA database included in our analysis. RCC, renal cell carcinoma.
