# Supplementary figures and images for: Subgroup-Independent Mapping of Renal Cell Carcinoma—Machine Learning Reveals Prognostic Mitochondrial Gene Signature Beyond Histopathologic Boundaries
Source: Front Oncol. 2021 Mar 15;11:621278. doi: 10.3389/fonc.2021.621278 (PMC8005734; doi:10.3389/fonc.2021.621278)

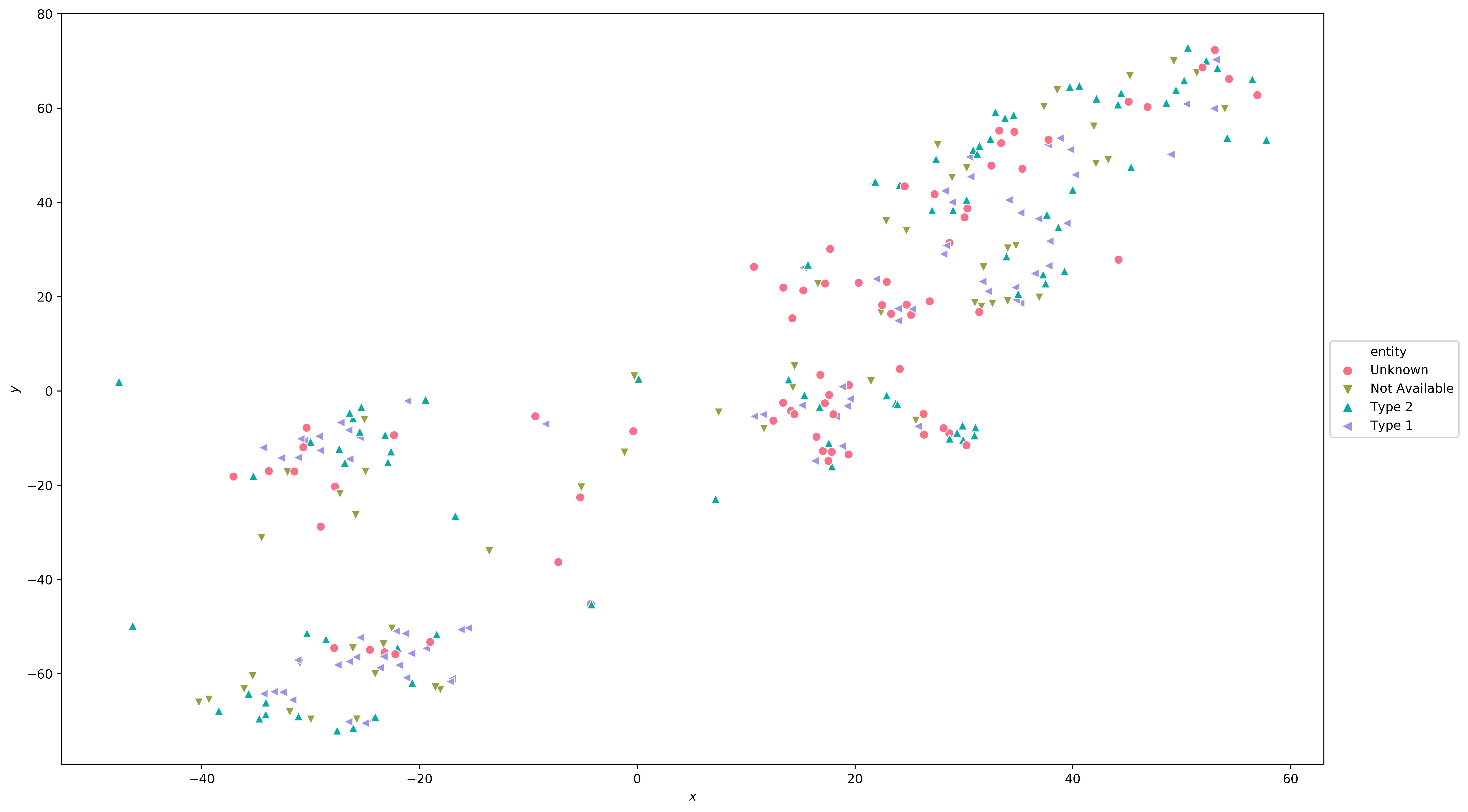

Supplement: Supplementary Figure 1 — Isolated illustration of pRCC samples from the pan-RCC clustering approach based on t-SNE plotting—with known affiliation to clinically established type 1 and type 2 pRCC subtypes. RCC, renal cell carcinoma; pRCC, papillary renal cell carcinoma. [file Image_1.JPEG]

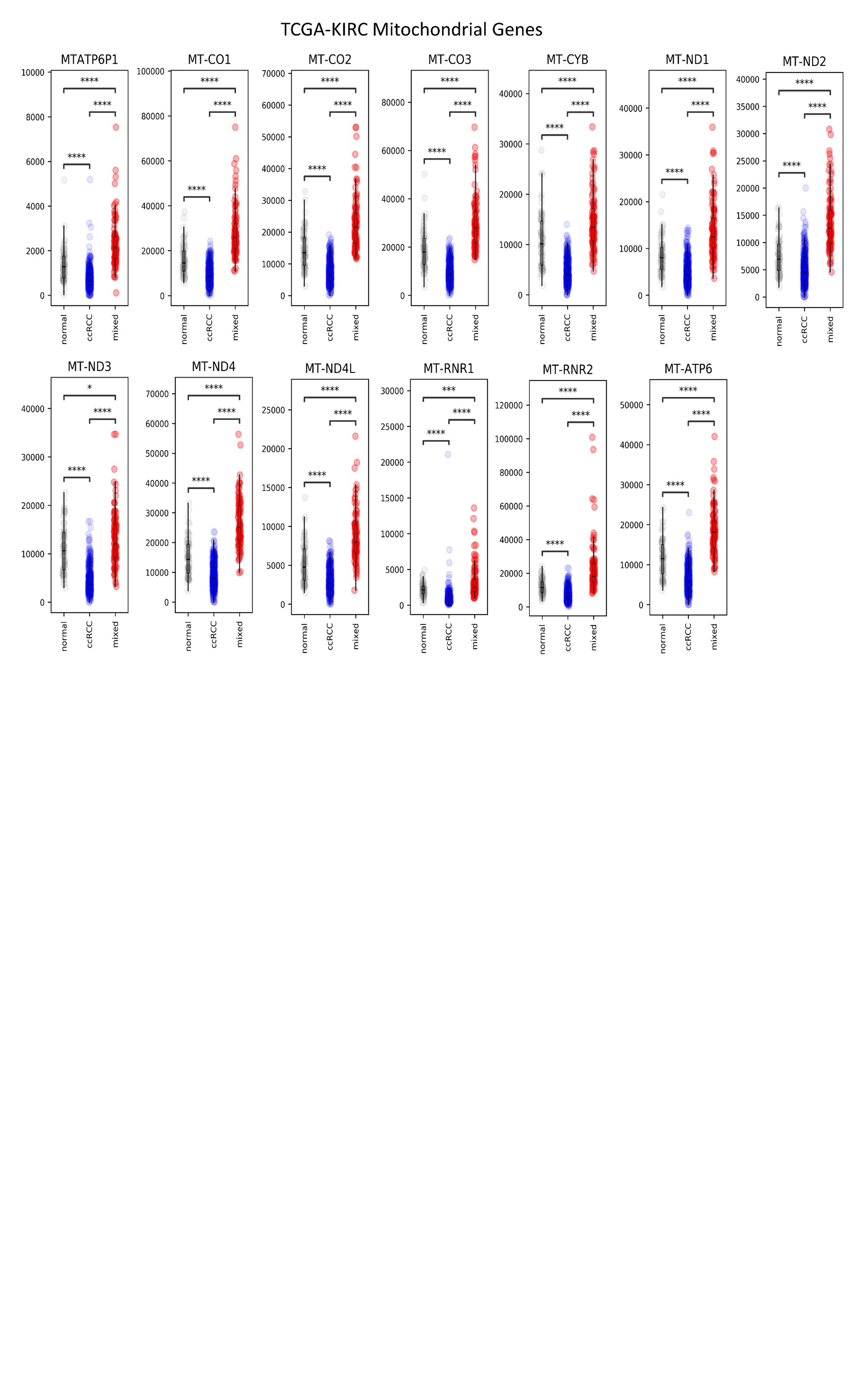

Supplement: Supplementary Figure 2 — Expression comparison between clear cell renal cell carcinomas outside (ccRCC) and inside (mixed) the mixed subgroup and respective normal tissue samples for mitochondrial genes identified by machine learning. ns, not significant. *p < 0.05, **p < 0.01, ***p < 0.001, ****p < 0.0001. [file Image_2.JPEG]

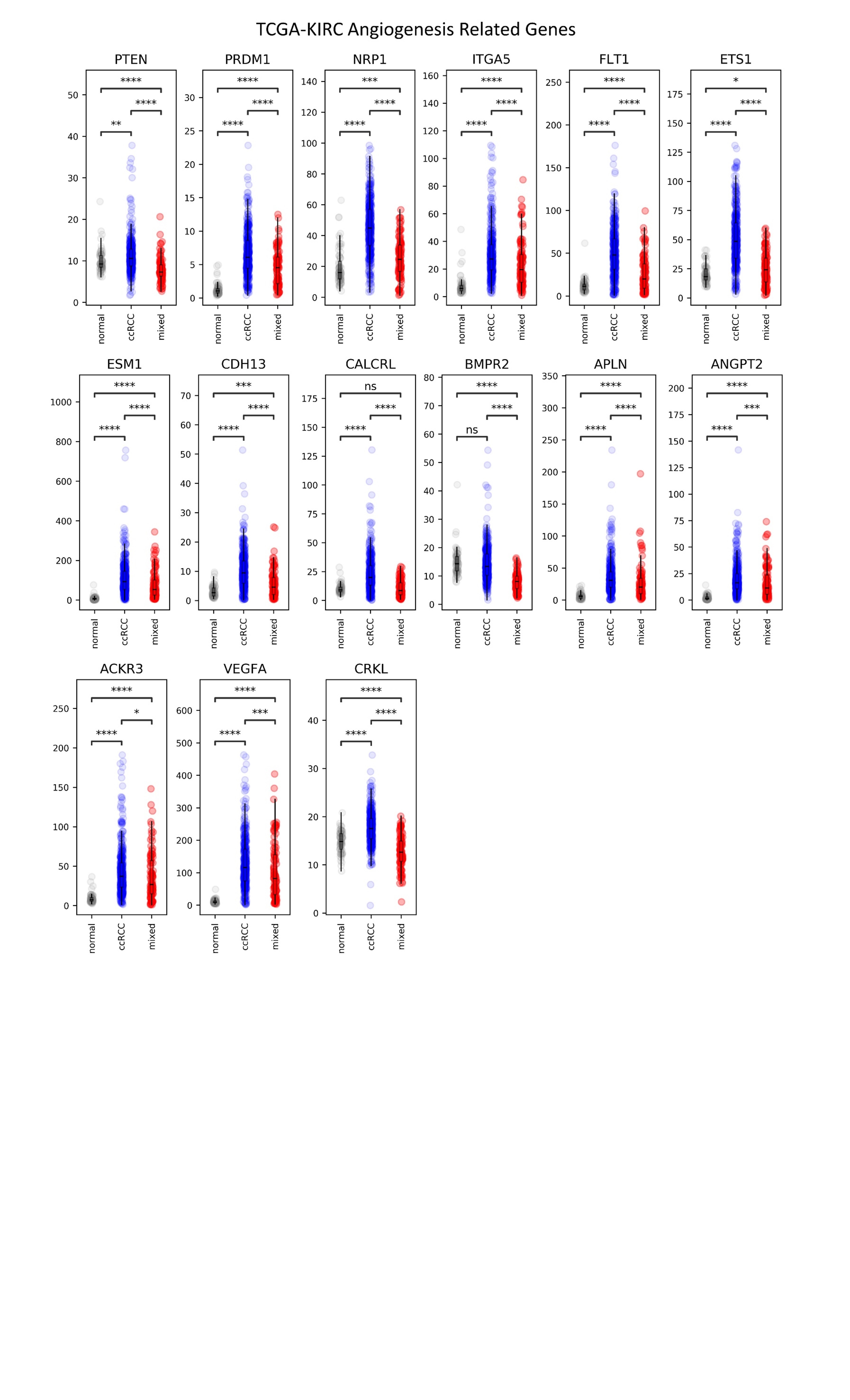

Supplement: Supplementary Figure 3 — Expression comparison between clear cell renal cell carcinomas outside (ccRCC) and inside (mixed) the mixed subgroup and respective normal tissue samples for angiogenesis genes identified by machine learning. ns, not significant. *p < 0.05, **p < 0.01, ***p < 0.001, ****p < 0.0001. [file Image_3.JPEG]

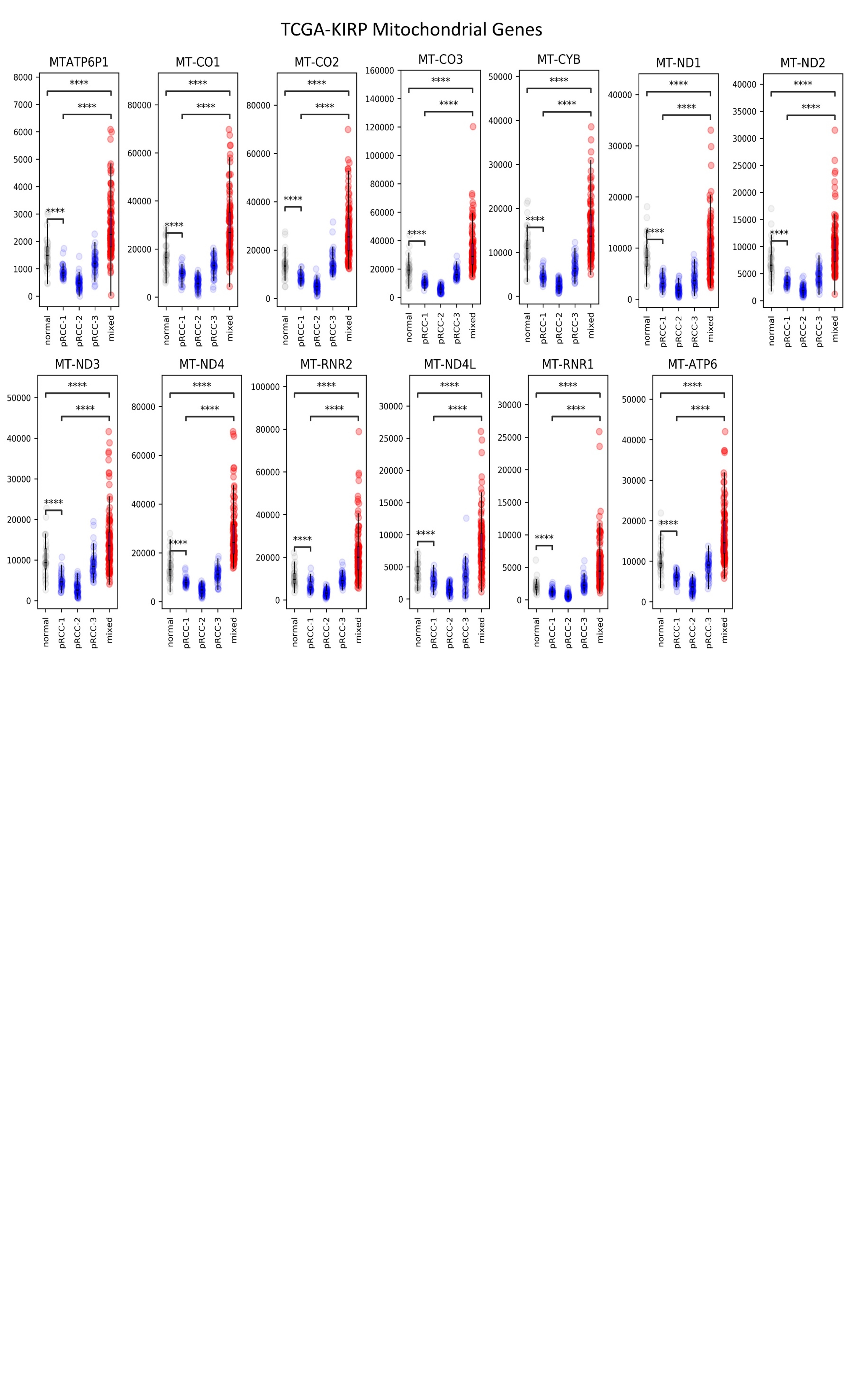

Supplement: Supplementary Figure 4 — Expression comparison between the identified papillary renal cell carcinoma cluster outside (pRCC 1 to 3) and inside (mixed) the mixed subgroup and respective normal tissue samples for mitochondrial genes identified by machine learning. ns, not significant. *p < 0.05, **p < 0.01, ***p < 0.001, ****p < 0.0001. [file Image_4.JPEG]

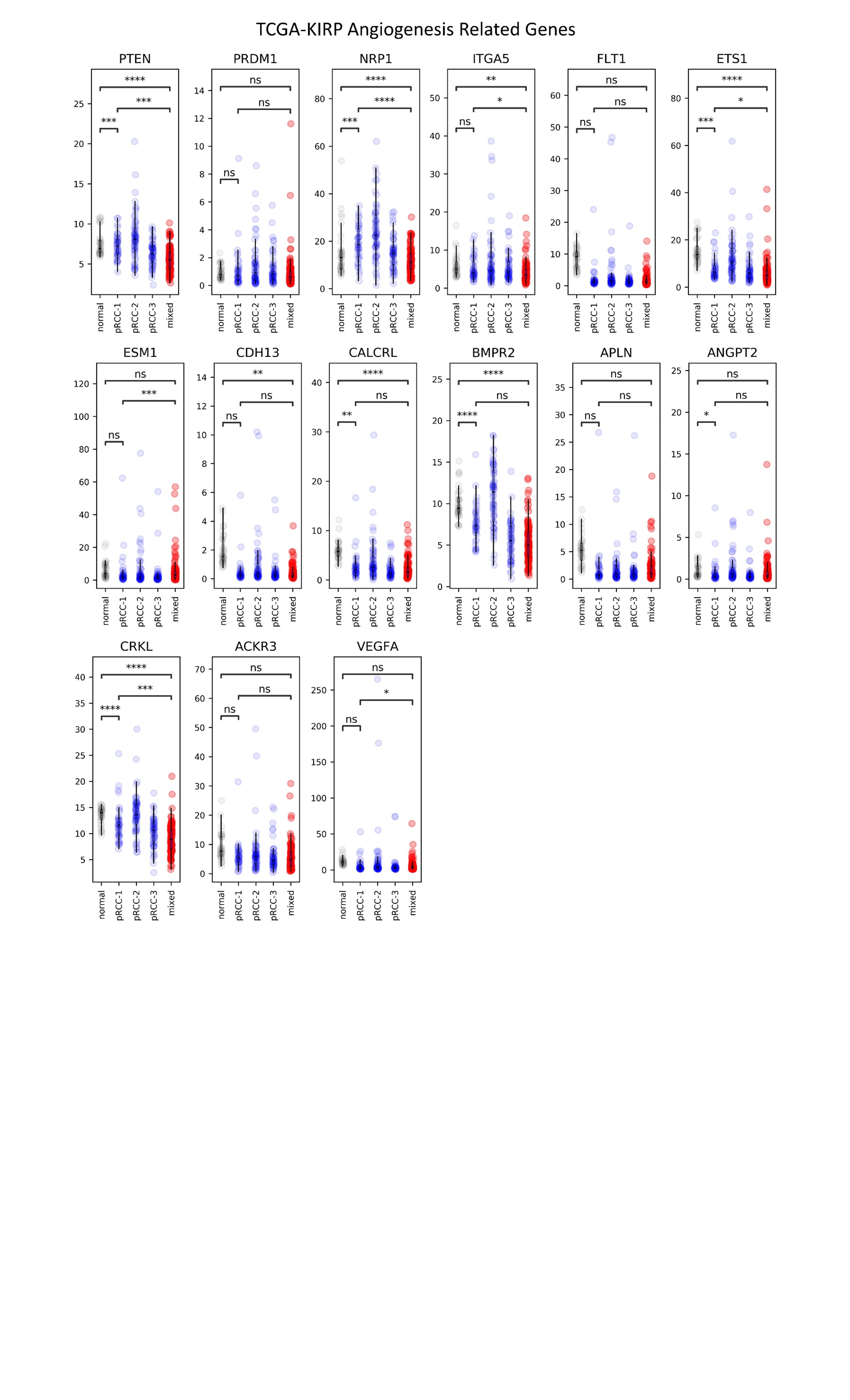

Supplement: Supplementary Figure 5 — Expression comparison between the identified papillary renal cell carcinoma cluster outside (pRCC 1 to 3) and inside (mixed) the mixed subgroup and respective normal tissue samples for angiogenesis genes identified by machine learning. ns, not significant. *p < 0.05, **p < 0.01, ***p < 0.001, ****p < 0.0001. [file Image_5.JPEG]

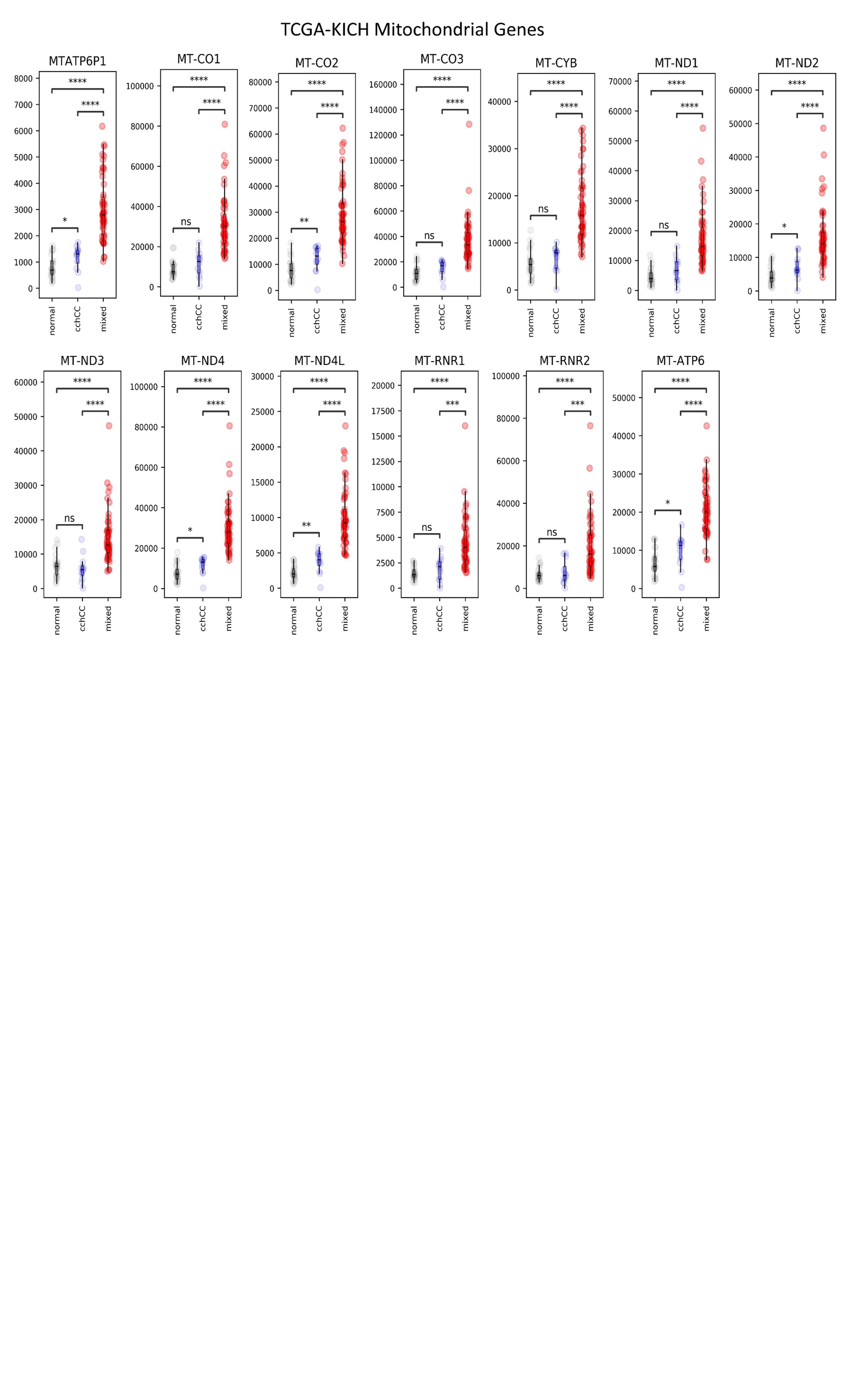

Supplement: Supplementary Figure 6 — Expression comparison between chromophobe renal cell carcinomas outside (chRCC) and inside (mixed) the mixed subgroup and respective normal tissue samples for mitochondrial genes identified by machine learning. ns, not significant. *p < 0.05, **p < 0.01, ***p < 0.001, ****p < 0.0001. [file Image_6.JPEG]

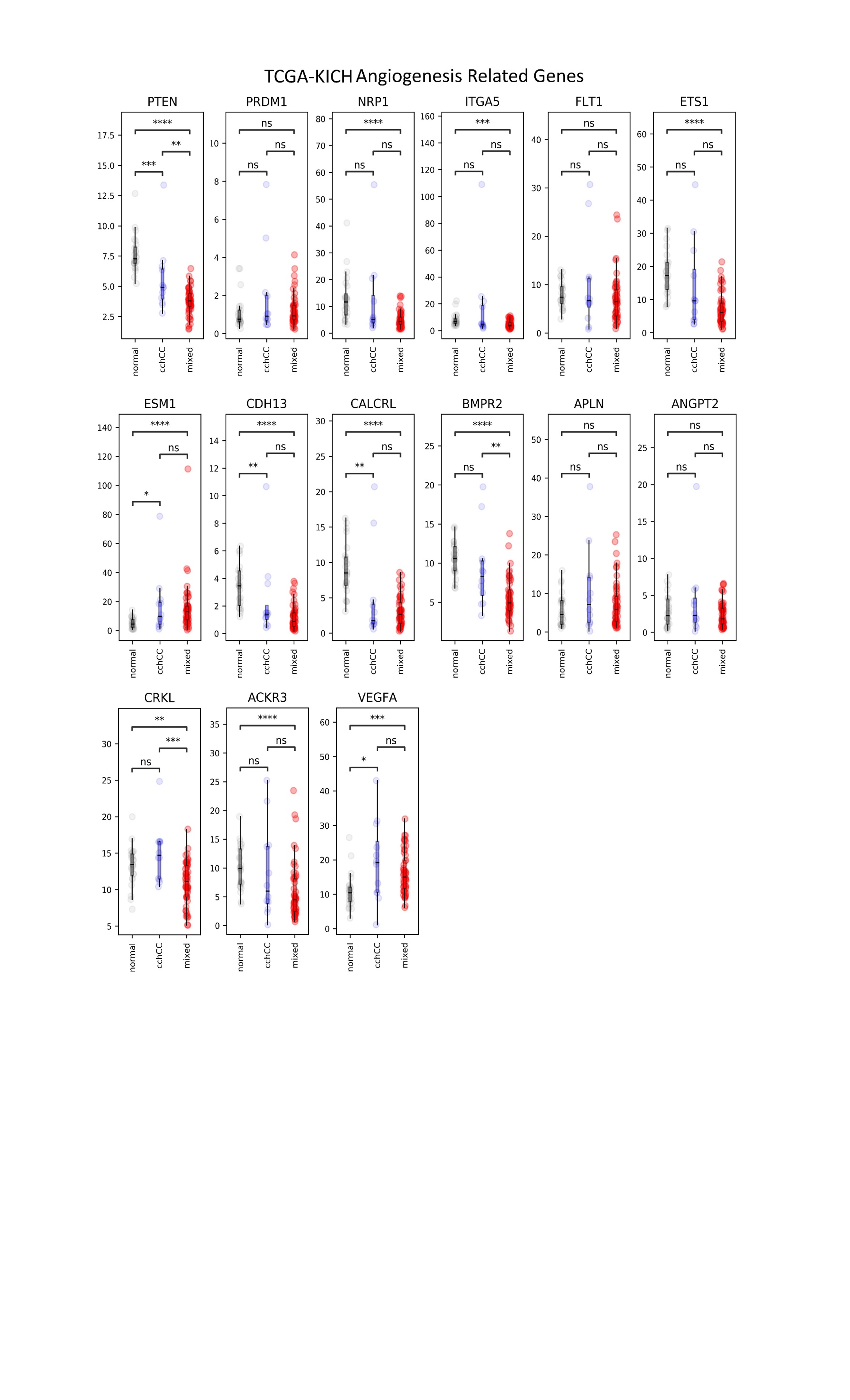

Supplement: Supplementary Figure 7 — Expression comparison between chromophobe renal cell carcinomas outside (chRCC) and inside (mixed) of mixed subgroup and respective normal tissue samples for angiogenesis genes identified by machine learning. ns, not significant. *p < 0.05, **p < 0.01, ***p < 0.001, ****p < 0.0001. [file Image_7.jpg]

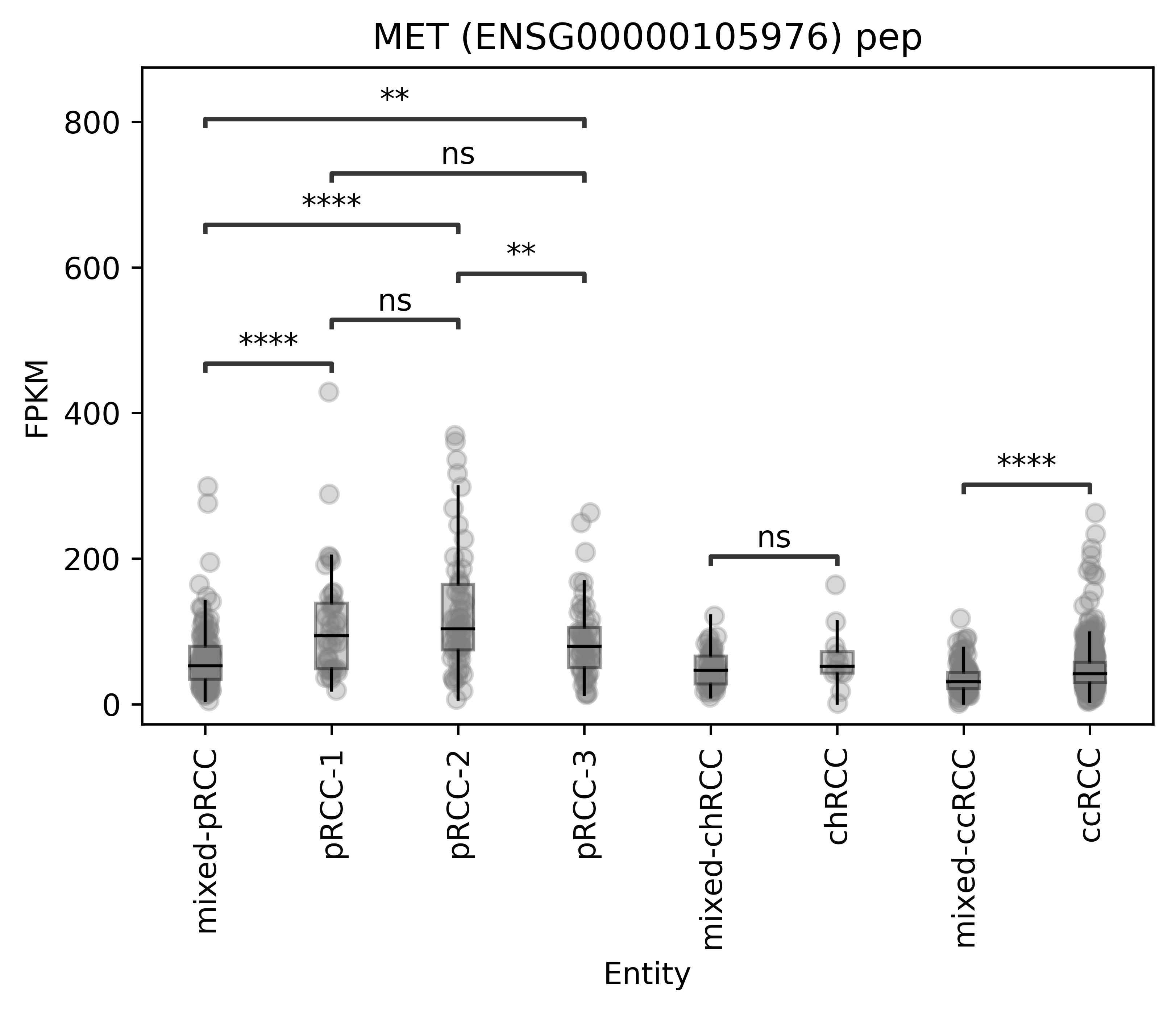

Supplement: Supplementary Figure 8 — Unprocessed FPKM values for c-MET within manually annotated clusters across RCC subgroups. ns, not significant. **p < 0.01, ****p < 0.0001. [file Image_8.JPEG]
